# Guideline-discordant dosing of direct-acting oral anticoagulants in the veterans health administration

**DOI:** 10.1186/s12913-021-07397-x

**Published:** 2021-12-18

**Authors:** Adam J. Rose, Jong Soo Lee, Dan R. Berlowitz, Weisong Liu, Avijit Mitra, Hong Yu

**Affiliations:** 1 Hebrew University School of Public Health, Ein Kerem Campus, Jerusalem, Israel; 2 School of Public Health, University of Massachusetts, Lowell, MA USA; 3 Edith Nourse Rogers VA Medical Center, Bedford, MA USA; 4 College of Information and Computer Science, University of Massachusetts, Amherst, MA USA

**Keywords:** Atrial fibrillation, Medication therapy management, Quality of health care, Anticoagulants

## Abstract

**Background:**

Clear guidelines exist to guide the dosing of direct-acting oral anticoagulants (DOACs). It is not known how consistently these guidelines are followed in practice.

**Methods:**

We studied patients from the Veterans Health Administration (VA) with non-valvular atrial fibrillation who received DOACs (dabigatran, rivaroxaban, apixaban) between 2010 and 2016. We used patient characteristics (age, creatinine, body mass) to identify which patients met guideline recommendations for low-dose therapy and which for full-dose therapy. We examined how often patient dosing was concordant with these recommendations. We examined variation in guideline-concordant dosing by site of care and over time. We examined patient-level predictors of guideline-concordant dosing using multivariable logistic models.

**Results:**

A total of 73,672 patients who were prescribed DOACS were included. Of 5837 patients who were recommended to receive low-dose therapy, 1331 (23%) received full-dose therapy instead. Of 67,935 patients recommended to receive full-dose therapy, 4079 (6%) received low-dose therapy instead. Sites varied widely on guideline discordant dosing; on inappropriate low-dose therapy, sites varied from 0 to 15%, while on inappropriate high-dose therapy, from 0 to 41%. Guideline discordant therapy decreased by about 20% in a relative sense over time, but its absolute numbers grew as DOAC therapy became more common. The most important patient-level predictors of receiving guideline-discordant therapy were older age and creatinine function being near the cutoff value.

**Conclusions:**

A substantial portion of DOAC prescriptions in the VA system are dosed contrary to clinical guidelines. This phenomenon varies widely across sites of care and has persisted over time.

**Supplementary Information:**

The online version contains supplementary material available at 10.1186/s12913-021-07397-x.

## Background

Since they were first approved by the US Food and Drug Administration (FDA) in 2010, direct-acting oral anticoagulants (DOACs) have become the most commonly-prescribed anticoagulants for non-valvular atrial fibrillation (NVAF) [1]. For each DOAC, FDA established clear criteria for which patients should receive full-dose therapy and which should receive low-dose therapy, based on the data from the clinical trials that led to their approval [2–4]. Relatively few studies, with relatively small sample sizes, have examined the extent to which these studies are being followed in practice [5, 6]. The issue of guideline-discordant dosing, especially of a potentially dangerous medication like an anticoagulant, is an important issue for quality of care.

We therefore used data from the US Veterans’ Health Administration (VA), the largest integrated health system in the United States, to examine guideline-concordant dosing of DOACs for patients with NVAF during the period from 2010 to 2016. We examined whether guideline-concordant dosing varies by site of care, whether it has improved over time, and also patient-level predictors of receiving guideline-discordant dosing. The results can help provide a sense of how common guideline-discordant DOAC dosing is in practice, and also help provide a roadmap for which patients are most likely to receive guideline-discordant dosing, and thus how best to address the issue.

## Methods

### Dataset

We used data from the VA Corporate Data Warehouse, a source that includes patient demographics, diagnosis codes, dates of service, laboratory test results, and medications dispensed. Using diagnosis codes, we identified all patients treated in the VA system with a diagnosis of NVAF between January 1, 2007, and December 30, 2016. Patients were considered to have NVAF if they had International Classification of Diseases, Clinical Modification, Version 9 (ICD-9-CM) code 427.31 or ICD-10-CM codes I48.xx, and did not have one of the ICD codes listed for valvular heart disease in Additional file 1. Additional details about this dataset and how we built it are available in our previous publication [1]. The study was approved by the Institutional Review Boards of the Bedford VA Medical Center and the University of Massachusetts Medical School, with a waiver of informed consent due to this being an analysis of an existing database. All study methods were conducted in accordance with the relevant guidelines and regulations.

### Direct-acting Oral anticoagulants

Among the population of patients with NVAF, we examined the receipt of DOACs between 2010 and 2016. The following DOACs were used in the VA during this period: dabigatran (starting in 2010), rivaroxaban (starting in 2012), and apixaban (starting in 2013). We required a minimum of 30 days of DOAC supply for study inclusion. Finally, we limited this study to VA patients who also were beneficiaries of fee-for-service Medicare, to ensure relatively complete data capture.

### Definitions of low-dose and full-dose direct-acting Oral anticoagulants

The major focus of this manuscript is to examine, among patients who received a DOAC, which ones received a full-dose DOAC and which received a low-dose DOAC. A full dose of dabigatran was defined as 150 mg, taken twice a day. For the purposes of this study, low-dose dabigatran was 75 mg, twice a day. These doses are mentioned in the official prescribing information for dabigatran [4]. Another dose of dabigatran approved by the FDA (110 mg) is not recommended by the VA pharmacy service for treatment of NVAF [7], and thus was not received by any VA patients.

A full dose of rivaroxaban was defined as 20 mg once a day, while low-dose rivaroxaban was defined as 15 mg once a day. These doses are mentioned in the official prescribing information for rivaroxaban [3]. A full dose of apixaban was defined as 5 mg twice a day, while low-dose apixaban was defined as 2.5 mg twice a day. These doses are mentioned in the official prescribing information for apixaban [2].

We excluded some patients to draw a clearer comparison between full-dose and low-dose DOACs. Patients who received more than one of the three DOACs were excluded from these analyses. Similarly, patients who received both full-dose and low-dose DOAC were excluded from this study, because they could not be unequivocally placed into either group.

### Patient-level variables

There were 130 VA Medical Centers (VAMC) in this analysis, each of which includes a hospital and several satellite outpatient clinics. Patients were assigned to one of these VAMCs based on the facility where they received their DOAC. Those few patients who received DOACs from more than one facility were excluded.

We characterized patients based on their age, sex, body mass index (BMI) at the time of the first DOAC prescription, and region of the US (Northeast, Midwest, West, and South). We characterized whether patients had a history of comorbid conditions contained within the CHADS-VASc stroke risk score [8]. These include heart failure, hypertension, vascular disease, diabetes, and prior stroke. We also identified patients who had prior episodes of major hemorrhage. We identified these conditions using ICD diagnosis codes, as listed in Additional file 1. We also calculated a count of Elixhauser comorbidities for each patient, using diagnostic codes reported as part of hospital and ambulatory encounters [9].

We calculated each patient’s estimated glomerular filtration rate (eGFR) from laboratory creatinine findings and other parameters, using the Modification of Diet in Renal Disease (MDRD) formula [10]. This formula was selected because of its compatibility with the creatinine assays used in the VA system throughout the study period, and because it is the basis for the automatic eGFR calculation provided to clinicians at most VA facilities. A recent publication has shown that MDRD is an acceptable choice to guide DOAC dosing decisions [11]. For patients with multiple creatinine values during the study period, we used the most recent value prior to the first DOAC fill.

### Analyses

We tabulated the number of unique patients who received each of the three DOACs, and separated them into recipients of full-dose and low-dose therapy. We also characterized each of these patients as meeting guidelines for low-dose or full-dose therapy, based on the information in the package inserts for each drug. Based on the FDA package insert for dabigatran [4] and VA recommendations [7], low-dose therapy should be offered to those with eGFR < 30 and full-dose therapy for all others. For rivaroxaban, low-dose therapy should be offered to those with eGFR < 50, and full-dose therapy for all others [3, 7].

Finally, for apixaban, low-dose therapy should be offered to those with two or more of the following three factors: age ≥ 80, body weight ≤ 60 kg, or serum creatinine ≥1.5 [2, 7]. Because the apixaban dosing criteria divide patients into mutually exclusive groups, it is not possible to enter these groups into a single regression analysis. We therefore created eight separate groups for recipients of apixaban – those meeting all 3 criteria to receive low-dose therapy, those meeting two of three criteria (three groups), those meeting one of three criteria (and therefore recommended for full-dose therapy, three groups), and those with no criteria.

We performed a multivariable logistic regression analysis among patients meeting criteria to receive full-dose therapy, to predict based on patient characteristics which patients would in fact receive low-dose therapy, contrary to FDA and VA guidance. We also performed similar regressions among patients meeting criteria to receive low-dose therapy, to predict based on patient characteristics which patients would receive guideline-discordant full-dose therapy. For recipients of apixaban, as discussed above, we performed these regressions for a total of eight groups of patients, defined by which of the criteria they met.

Finally, a considerable number of patients were excluded from the study due to having received multiple DOACs or multiple different doses. To better understand what impact these exclusions may have had on the study results, we examined a subset of these excluded patients – those who had received rivaroxaban. For those who received rivaroxaban and another DOAC, we examined which medication they received first and whether their rivaroxaban dose was guideline-concordant based on eGFR. For those who received both doses of rivaroxaban, we calculated how many received the higher dose first and how many the lower dose first, and of those, which one was the guideline-concordant dose. All analyses were conducted with R, version 3.6.3 (R Foundation for Statistical Computing, Vienna, Austria).

## Results

### Exclusions and final study sample

A total of 109,989 VA patients received a DOAC during the period between 2010 and 2016 and had a diagnostic code for NVAF. Of these, 2256 were excluded because they received less than 30 days’ total supply, and 15,134 were excluded because they also had a diagnostic code for venous thromboembolism, complicating the question of why they were receiving anticoagulation. We excluded 12,695 because of one or more of the following: they received more than one type of DOAC, they received more than one dose of DOAC, and/or they received DOAC prescriptions from more than one VA medical center. We excluded 2705 patients because of missing demographic data and 3437 because of other missing data, such as having no creatinine values recorded. The final sample that we analyzed here was therefore 73,672 patients.

The patient-level characteristics of the 73,672 patients who were included in our main study are summarized in Table 1. As is usual for a VA population, the patients were mostly male (98%) and mostly of White race (86%). This population of older patients with NVAF had a high burden of comorbid illness. For example, 41% had 3–4 Elixhauser comorbid conditions, and 29% had 5 or more. Regarding renal function, only 2% had an eGFR below 30, while 9% had an eGFR of 30–44, and 15% had an eGFR of 45–59.

**Table 1:** Characteristics of 73,672 VA patients prescribed direct-acting oral anticoagulants between 2011 and 2016

Characteristic	Percentage
**Age (yrs)**	
< 65	16%
65–74	44%
75–84	27%
> 85	13%
**Sex**	
Female	2%
Male	98%
**Race**	
White	86%
Black	8%
Other	6%
**Geographic Region**	
Northeast	14%
Mid-West	24%
West (Incl. Pacific)	22%
South	40%
**Body Mass Index (kg/m**^**2**^**)**	
< 25	19%
25–29.9	33%
30–34.9	26%
> = 35	28%
**Comorbid Conditions**	
Heart Failure	15%
Hypertension	73%
Vascular Disease	17%
Diabetes	48%
Prior Bleeding	4%
Prior Stroke	7%
**CHA**_**2**_**DS**_**2**_**-VASc Score (%)**	
0–1	11%
2–4	77%
5–9	13%
**eGFR Categories (%), in units of mL/min/1.73 m**^**2**^	
< 30	2%
30–44	9%
45–59	15%
> 60	74%
**Elixhauser Comorbidities**	
0–2	30%
3–4	41%
> = 5	29%

Because of rounding, percentages may not sum to 100%

### Guideline-discordant dosing

A considerable proportion of patients received guideline-discordant doses of DOACs. Across the entire study period, of the 5837 patients recommended to receive low-dose therapy, 1331 received full-dose therapy instead (23%). Of these, the most in absolute terms were receiving inappropriately high doses of rivaroxaban (661 patients), followed by apixaban (497 patients). Of the 67,935 patients recommended to receive full-dose therapy, 4079 received low-dose therapy instead (6%). The largest absolute contribution to this was from apixaban (2376 patients), followed by rivaroxaban (1221 patients).

**Fig. 1:**
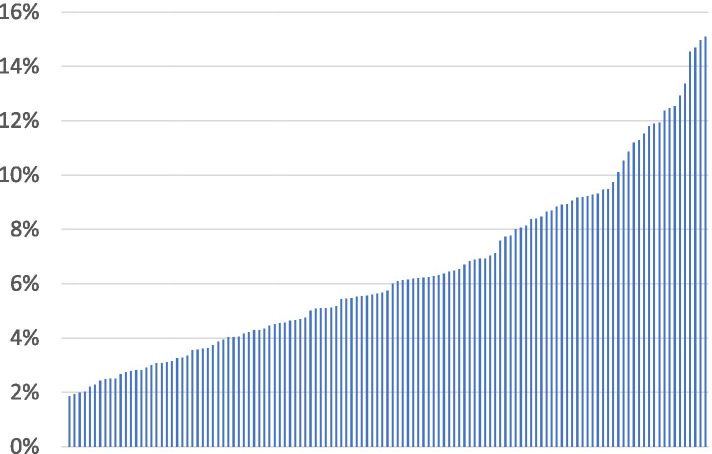
Site-variation in guideline-discordant DOAC prescribing, 2010–2016. Includes prescriptions for dabigatran, rivaroxaban, and apixaban. Proportion of patients appropriate for full-dose DOAC therapy who received low-dose therapy instead, by site. Among 126 sites with at least 100 patients in the denominator

**Fig. 2:**
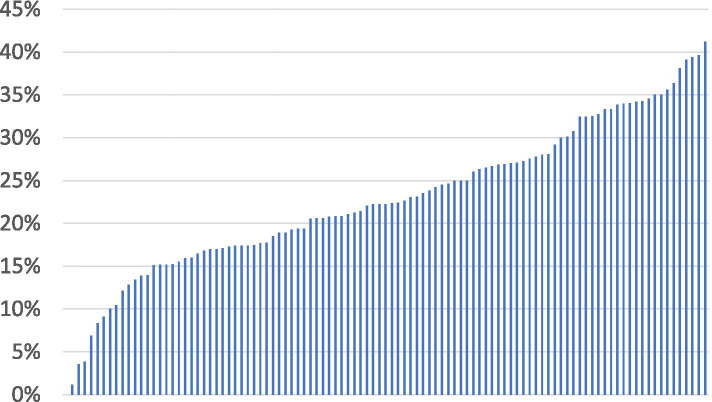
Site-variation in guideline-discordant DOAC prescribing, 2010–2016. Includes prescriptions for dabigatran, rivaroxaban, and apixaban. Proportion of patients appropriate for low-dose DOAC therapy who received full-dose therapy instead, by site. Among 103 sites with at least 20 patients in the denominator

This use of guideline-discordant dosing was not uniform by site of care (Fig. 1). Among the 126 sites with at least 100 patients recommended for full-dose DOAC therapy throughout the study period, sites varied from 0% of patients receiving inappropriately low doses to as high as 15%. Similarly, among the 103 sites with at least 20 patients recommended for low-dose DOAC therapy throughout the study period (Fig. 2), sites varied from 0% of patients receiving inappropriately full doses to as high as 41%.

Our further analyses focus on guideline-discordant low-dose therapy, because it was a more common phenomenon in absolute terms than guideline-discordant full-dose therapy. Results regarding guideline-discordant full-dose therapy are found in Additional file 2, and are not discussed here.

Table 2 shows trends over time in the use of low-dose DOAC therapy among patients who met clinical criteria for full-dose therapy. While some cells have small numbers, especially in the years 2010 and 2011, we see several findings. First, the absolute rate of guideline-discordant low-dose dabigatran use is lower than the other medications – possibly because guidelines recommend low-dose dabigatran for a much smaller group of patients, namely those with eGFR < 30. Second, the proportion of patients receiving guideline-discordant low-dose DOACs decreased somewhat, for each medication, over time, in relative terms, by about 20%. In absolute terms, many more patients received inappropriate low-dose therapy in each successive year, as the absolute number of patients receiving DOAC therapy increased over time. Third, by the end of the study period, the use of guideline-discordant low-dose apixaban was the most common among any of the DOACs, both in absolute and relative terms.

**Table 2:** Trends over time in use of low-dose DOAC therapy for non-valvular atrial fibrillation, among patients meeting criteria for full-dose therapy

Year	Should receive full-dose DOAC	Received Low-Dose DOAC		Should receive full-dose Dabi	Received Low-Dose Dabi		Should receive full-dose Riva	Received Low-Dose Riva		Should receive full-dose Apix	Received Low-Dose Apix	
2010	9	2	22%	9	2	22%						
2011	1635	37	2%	1635	37	2%						
2012	2860	79	3%	2814	76	3%	46	3	7%			
2013	3958	158	4%	3383	92	3%	507	50	10%	68	16	24%
2014	9791	626	6%	5431	104	2%	2904	289	10%	1456	233	16%
2015	18,345	1206	7%	5530	73	1%	5424	389	7%	7391	744	10%
2016	31,327	1971	6%	7344	98	1%	7946	490	6%	16,037	1383	9%

Dabi – dabigatran

Riva – rivaroxaban

Apix - apixaban

### Patient-level predictors of inappropriate low-dose therapy

We also looked at the patient-level predictors of receiving a low-dose DOAC, as opposed to full-dose, among patients recommended for full-dose therapy. For these models, we looked at each medication separately, since each medication has its own criteria for low-dose therapy [2–4]. Table 3 shows the patient-level predictors of receiving guideline-discordant low-dose dabigatran. The strongest predictor of guideline-discordant low-dose dabigatran therapy was kidney function just above the cutoff value (eGFR 30–39, AOR 18.16, *p* <  0.001), with less severe kidney disfunction also a relatively important predictor. Older patients were also more likely to receive guideline-discordant low-dose therapy (age 80+ AOR 6.41 compared to age ≤ 69, p <  0.001). Patients in the South were more likely to receive guideline-discordant low-dose therapy, compared to patients in the Northeast (OR 1.59, *p* = 0.006). Overweight and obesity also were significantly associated with receiving guideline-discordant low-dose therapy.

**Table 3:** Patient-level factors associated with receiving low-dose dabigatran from the Veterans Health Administration, as compared to receiving full-dose, among those who met clinical criteria to receive full-dose dabigatran for non-valvular atrial fibrillation (*n* = 26,146)

Characteristic	Low-Dose (*n* = 482)	Full-Dose (*n* = 25,664)	Adjusted Odds Ratio^a^ to Receive Low-dose DOAC	95% Confidence Interval	*p*-value
**Age (yrs)**					
≤ 69	18.0%	55.7%	REF		
70–74	10.4%	20.5%	1.43	(0.98, 2.06)	0.06
75–79	9.5%	10.8%	1.81	(1.20, 2.69)	0.004
> 80	62.0%	12.9%	6.41	(4.68, 8.84)	< 0.001
**Gender**					
Female	2.7%	1.6%	1.38	(0.68, 2.55)	0.34
Male	97.3%	98.4%	REF		
**Race**					
White	85.3%	86.6%	REF		
Black	7.7%	7.2%	1.28	(0.86, 1.87)	0.21
Other	7.1%	6.2%	1.06	(0.68, 1.59)	0.80
**Geographic Region**					
Northeast	12.0%	12.7%	REF		
Midwest	21.4%	23.9%	1.19	(0.83, 1.72)	0.34
West	21.0%	23.8%	1.25	(0.88, 1.81)	0.22
South	45.6%	39.6%	1.59	(1.16, 2.21)	0.004
**BMI (kg/m**^**2**^**)**					
< 25	28.4%	14.6%	REF		
25–29.9	35.7%	30.6%	1.32	(1.02, 1.73)	0.04
30–34.9	21.4%	27.5%	1.80	(1.31, 2.46)	< 0.001
≥ 35	11.2%	25.8%	1.52	(1.02, 2.26)	0.04
**Key Comorbid Conditions**^b^					
Heart Failure	20.3%	13.6%	1.36	(1.03, 1.79)	0.03
Hypertension	77.8%	71.6%	1.09	(0.83, 1.44)	0.55
Vascular Disease	15.4%	13.2%	1.15	(0.86, 1.53)	0.34
Diabetes	44.2%	41.4%	1.24	(0.98, 1.57)	0.07
Prior Bleeding	4.6%	3.1%	1.20	(0.73, 1.90)	0.45
Stroke	8.3%	5.9%	1.04	(0.71, 1.47)	0.85
**eGFR Categories (%), in units of mL/min/1.73 m**^**2**^					
30–39	28.0%	1.9%	18.16	(13.05, 25.27)	< 0.001
40–49	23.7%	4.6%	6.96	(5.05, 9.58)	< 0.001
50–59	17.4%	7.9%	3.36	(2.42, 4.64)	< 0.001
> 60	30.9%	85.6%	REF		
**Elixhauser Comorbidities**					
0–2	25.9%	30.0%	REF		
3–4	34.6%	39.1%	0.94	(0.71, 1.26)	0.69
≥ 5	30.5%	25.1%	1.35	(0.95, 1.91)	0.10

Model c-statistic: 0.85

^a^Adjusted for all the other variables in the table

^b^For each condition, the reference category is patients without the condition

Table 4 shows patient-level predictors of receiving guideline-discordant low-dose rivaroxaban. Again, we found that kidney function just above the cutoff level (eGFR 50–59) was associated with a greater likelihood of receiving guideline-discordant low-dose therapy, compared to eGFR 60+ (AOR 8.40, *p* <  0.001). Older patients were more likely to receive guideline-discordant low-dose therapy (age 80+ AOR 5.29 compared to age ≤ 69, *p* <  0.001), as were women (AOR 1.71, *p* = 0.02). Unlike dabigatran, there were no clear regional differences for this medication. As with dabigatran, overweight and obese were associated with a higher likelihood of receiving guideline-discordant low-dose therapy (AOR 2.53 for those with BMI ≥ 35, p <  0.001). Patients with more comorbid conditions were also more likely to receive a guideline-discordant low dose. For example, having 5 or more Elixhauser comorbidities was associated with AOR of 1.76, compared to 0–2 (p <  0.001).

**Table 4:** Patient-level factors associated with receiving low-dose rivaroxaban from the Veterans Health Administration, as compared to receiving full-dose, among those who met clinical criteria to receive full-dose rivaroxaban for non-valvular atrial fibrillation (*n* = 16,831)

Characteristic	Low-Dose (*n* = 1225)	Full-Dose (*n* = 15,606)	Adjusted Odds Ratio^a^ to Receive Low-dose DOAC	95% Confidence Interval	p-value
**Age (yrs)**					
≤ 69	22.9%	53.8%	REF		
70–74	16.1%	22.3%	1.61	(1.32, 1.97)	< 0.001
75–79	16.7%	11.8%	2.80	(2.26, 3.45)	< 0.001
> 80	44.2%	12.1%	5.77	(4.78, 6.97)	< 0.001
**Gender**					
Female	2.0%	1.6%	1.71	(1.06, 2.65)	0.02
Male	98.0%	98.4%	REF		
**Race**					
White	86.4%	85.2%	REF		
Black	7.0%	8.9%	0.99	(0.76, 1.28)	0.93
Other	6.5%	5.9%	1.21	(0.91, 1.57)	0.18
**Geographic Region**					
Northeast	15.6%	13.7%	REF		
Midwest	26.8%	25.8%	1.14	(0.91, 1.42)	0.25
West	20.0%	19.8%	1.14	(0.90, 1.44)	0.28
South	37.6%	40.7%	0.98	(0.80, 1.21)	0.85
**BMI (kg/m**^**2**^**)**					
< 25	14.6%	12.5%	REF		
25–29.9	35.1%	30.6%	1.51	(1.22, 1.87)	< 0.001
30–34.9	27.9%	28.8%	2.26	(1.80, 2.85)	< 0.001
≥ 35	20.1%	26.3%	2.53	(1.97, 3.25)	< 0.001
**Key Comorbid Conditions**^b^					
Heart Failure	19.4%	14.1%	1.48	(1.23, 1.78)	< 0.001
Hypertension	78.4%	72.3%	1.09	(0.91, 1.32)	0.35
Vascular Disease	17.1%	17.5%	0.88	(0.73, 1.06)	0.18
Diabetes	51.8%	48.5%	1.05	(0.90, 1.23)	0.52
Prior Bleeding	3.4%	3.5%	0.88	(0.61, 1.25)	0.50
Stroke	8.5%	6.5%	1.27	(0.99, 1.61)	0.058
**eGFR Categories (%), in units of mL/min/1.73 m**^**2**^					
50–59	48.6%	7.6%	8.40	(7.16, 9.85)	< 0.001
> 60	51.4%	92.4%	REF		
**Elixhauser Comorbidities**					
0–2	22.2%	28.3%	REF		
3–4	38.3%	38.3%	1.27	(1.05, 1.53)	0.02
≥ 5	32.6%	27.2%	1.80	(1.43, 2.27)	< 0.001

Model c-statistic: 0.81

^a^Adjusted for all the other variables in the table

^b^For each condition, the reference category is patients without the condition

Finally, for apixaban (Tables 5, 6, 7 and 8), low-dose therapy is indicated for people who have at least two of the following: age ≥ 80, body weight < 60 kg, or serum creatinine ≥1.5 [2]. As discussed above, we conducted four analyses of the patients who did not qualify for a low dose: for those with none of the three criteria for low-dose therapy, and for those with only one criterion (three analyses). In all four analyses, we found that age, creatinine, and body mass were important predictors for who would receive guideline-discordant low-dose therapy. For example, in Table 6, we focus on those patients whose sole “abnormal” parameter was being age 80 and older, but who had normal creatinine and body mass more than 60 kg. Even among this population of oldest-old patients, age was still a strong predictor of guideline-discordant low dose (AOR 3.37 for age 90+, compared to age 80–84, *p* <  0.001). Body weight of 61–69 kg, which is just above the weight cutoff, was also associated with greater odds of receiving a guideline-discordant low dose (AOR 2.62 compared to 80+ kg, p <  0.001). Finally, creatinine of 1.30–1.49, although not sufficient to warrant low-dose therapy per the guidelines, was also associated with greater odds of receiving a guideline-discordant low dose (AOR 5.42 compared to creatinine less than 1, p <  0.001). Generally similar findings were seen in the other analyses (Tables 5, 7, and 8).

**Table 5:** Patient-level factors associated with receiving low-dose apixaban from the Veterans Health Administration, as compared to receiving full-dose, among those who met clinical criteria to receive full-dose apixaban for non-valvular atrial fibrillation. This table includes patients with none of the three factors suggesting a need for low-dose therapy (i.e., age < 80, serum creatinine < 1.5, and body mass > 60 kg, *n* = 13,162)

Characteristic	Low-Dose (*n* = 262)	Full-Dose (n = 12,900)	Adjusted Odds Ratio^a^ to Receive Low-dose DOAC	95% Confidence Interval	p-value
**Age (yrs)**					
≤ 69	26.0%	44.1%	REF		
70–74	27.1%	28.0%	1.86	(1.27, 2.72)	< 0.001
75–79	46.9%	27.9%	2.83	(1.99, 4.07)	0.001
**Gender**					
Female	0.8%	1.6%	0.60	(0.10, 1.94)	0.48
Male	99.2%	98.4%	REF		
**Race**					
White	87.4%	86.9%	REF		
Black	6.9%	7.4%	0.97	(0.54, 1.62)	0.91
Other	5.7%	5.8%	0.91	(0.46, 1.62)	0.77
**Geographic Region**					
Northeast	11.5%	14.1%	REF		
Midwest	22.5%	25.2%	1.30	(0.80, 2.18)	0.30
West	23.3%	20.9%	1.07	(0.64, 1.83)	0.80
South	42.7%	39.8%	1.40	(0.89, 2.27)	0.16
**Weight, kg**					
61–69	12.6%	4.8%	3.14	(2.00, 4.79)	< 0.001
70–79	17.9%	11.5%	1.75	(1.19, 2.52)	0.003
80+	69.5%	83.8%	REF		
**Key Comorbid Conditions**^b^					
Heart Failure	19.8%	14.7%	1.44	(0.99, 2.05)	0.050
Hypertension	71.8%	73.1%	0.85	(0.58, 1.26)	0.41
Vascular Disease	24.4%	22.2%	1.02	(0.72, 1.43)	0.91
Diabetes	58.0%	53.2%	1.29	(0.94, 1.80)	0.12
Prior Bleeding	4.2%	4.6%	0.84	(0.40, 1.58)	0.63
Stroke	5.3%	7.3%	0.68	(0.36, 1.18)	0.20
**Creatinine Categories (%), in mg/dL**					
< 1	30.5%	36.5%	REF		
1.0–1.29	29.0%	29.3%	1.21	(0.87, 1.69)	0.26
1.3–1.49	25.6%	15.6%	1.79	(1.26, 2.55)	0.001
**Elixhauser Comorbidities**					
0–2	25.6%	27.2%	REF		
3–4	32.8%	38.8%	0.81	(0.54, 1.22)	0.31
≥ 5	34.4%	28.2%	1.22	(0.76, 1.95)	0.41

Model c-statistic: 0.69

^a^Adjusted for all the other variables in the table

^b^For each condition, the reference category is patients without the condition

**Table 6:** Patient-level factors associated with receiving low-dose apixaban from the Veterans Health Administration, as compared to receiving full-dose, among those who met clinical criteria to receive full-dose apixaban for non-valvular atrial fibrillation. This table includes patients with only age suggesting a need for low-dose therapy (i.e., age ≥ 80, serum creatinine < 1.5, and body mass > 60 kg, *n* = 8611)

Characteristic	Low-Dose (*n* = 1773)	Full-Dose (*n* = 6838)	Adjusted Odds Ratio^a^ to Receive Low-dose DOAC	95% Confidence Interval	p-value
**Age (yrs)**					
80–84	34.9%	53.7%	REF		
85–89	36.3%	34.1%	1.61	(1.39, 1.86)	< 0.001
90+	28.9%	12.2%	3.37	(2.84, 3.99)	< 0.001
**Gender**					
Female	1.6%	1.2%	0.94	(0.52, 1.62)	0.82
Male	98.4%	98.8%	REF		
**Race**					
White	90.6%	91.5%	REF		
Black	3.2%	3.2%	0.83	(0.58, 1.17)	0.30
Other	6.1%	5.3%	1.44	(1.10, 1.87)	0.007
**Geographic Region**					
Northeast	17.9%	17.9%	REF		
Midwest	21.8%	24.3%	0.96	(0.78, 1.17)	0.66
West	20.3%	21.7%	0.90	(0.73, 1.10)	0.30
South	40.0%	36.2%	1.10	(0.92, 1.33)	0.29
**Weight, kg**					
61–69	26.3%	12.2%	2.62	(2.21, 3.10)	< 0.001
70–79	27.9%	26.7%	1.27	(1.09, 1.48)	0.002
80+	45.7%	61.1%	REF		
**Key Comorbid Conditions**^b^					
Heart Failure	14.4%	11.4%	1.02	(0.83, 1.23)	0.88
Hypertension	76.4%	72.9%	1.01	(0.85, 1.20)	0.91
Vascular Disease	17.0%	16.2%	0.98	(0.82, 1.16)	0.79
Diabetes	47.7%	45.7%	0.98	(0.85, 1.13)	0.79
Prior Bleeding	4.5%	3.6%	1.04	(0.76, 1.41)	0.81
Stroke	8.0%	7.7%	0.89	(0.70, 1.12)	0.31
**Creatinine Categories (%), in mg/dL**					
< 1	17.9%	32.8%	REF		
1.0–1.29	29.4%	32.6%	1.77	(1.50, 2.09)	< 0.001
1.3–1.49	41.5%	15.6%	5.42	(4.60, 6.40)	< 0.001
**Elixhauser Comorbidities**					
0–2	29.3%	34.6%	REF		
3–4	41.0%	39.8%	1.36	(1.15, 1.61)	< 0.001
≥ 5	21.0%	17.3%	1.57	(1.26, 1.95)	< 0.001

Model c statistic: 0.75

^a^Adjusted for all the other variables in the table

^b^For each condition, the reference category is patients without the condition

**Table 7:** Patient-level factors associated with receiving low-dose apixaban from the Veterans Health Administration, as compared to receiving full-dose, among those who met clinical criteria to receive full-dose apixaban for non-valvular atrial fibrillation. This table includes patients with only body mass suggesting a need for low-dose therapy (i.e., age < 80, serum creatinine < 1.5, and body mass ≤ 60 kg, *n* = 242)

Characteristic	Low-Dose (*n* = 28)	Full-Dose (*n* = 214)	Adjusted Odds Ratio^a^ to Receive Low-dose DOAC	95% Confidence Interval	*p*-value
**Age (yrs)**					
≤ 69	17.9%	32.7%	REF		
70–74	17.9%	31.8%	1.56	(0.34, 7.71)	0.57
75–79	64.3%	35.5%	3.31	(0.94, 14.33)	0.08
**Gender**					
Female	17.9%	9.8%	1.84	(0.43, 6.70)	0.37
Male	82.1%	90.2%	REF		
**Race**					
White	82.1%	81.8%	REF		
Black	10.7%	10.7%	0.98	(0.19, 3.80)	0.98
Other	7.1%	7.5%	0.91	(0.12, 4.47)	0.92
**Geographic Region**					
Northeast	7.1%	15.0%	REF		
Midwest	14.3%	24.3%	1.22	(0.20, 10.09)	0.84
West	28.6%	21.0%	2.43	(0.43, 19.88)	0.34
South	50.0%	39.7%	1.34	(0.27, 10.13)	0.74
**Weight, kg**					
< 50	28.6%	14.0%	2.87	(0.92, 8.64)	0.061
50–59	71.4%	86.0%	REF		
**Key Comorbid Conditions**^b^					
Heart Failure	21.4%	20.1%	1.25	(0.29, 5.03)	0.76
Hypertension	67.9%	62.1%	1.03	(0.29, 4.07)	0.97
Vascular Disease	14.3%	22.4%	0.89	(0.22, 3.14)	0.87
Diabetes	50.0%	41.1%	1.49	(0.49, 4.68)	0.48
Prior Bleeding	0.0%	4.7%	0	NA	NA
Stroke	7.1%	13.6%	0.21	(0.01, 1.40)	0.18
**Creatinine Categories (%), in mg/dL**					
< 1	67.9%	61.2%	REF		
1.0–1.29	7.1%	14.0%	0.68	(0.09, 3.04)	0.64
1.3–1.49	10.7%	9.3%	0.79	(0.10, 4.16)	0.79
**Elixhauser Comorbidities**					
0–2	14.3%	18.7%	REF		
3–4	35.7%	35.0%	1.83	(0.36, 11.34)	0.48
≥ 5	39.3%	41.6%	1.44	(0.21, 10.79)	0.71

Model c statistic: 0.75

^a^Adjusted for all the other variables in the table

^b^For each condition, the reference category is patients without the condition

**Table 8:** Patient-level factors associated with receiving low-dose apixaban from the Veterans Health Administration, as compared to receiving full-dose, among those who met clinical criteria to receive full-dose apixaban for non-valvular atrial fibrillation. This table includes patients with only creatinine suggesting a need for low-dose therapy (i.e., age < 80, serum creatinine ≥1.5, and body mass > 60 kg, *n* = 2397)

Characteristic	Low-Dose (*n* = 313)	Full-Dose (*n* = 2624)	Adjusted Odds Ratio^a^ to Receive Low-dose DOAC	95% Confidence Interval	p-value
**Age (yrs)**					
≤ 69	31.6%	41.0%	REF		
70–74	24.6%	31.7%	1.01	(0.72, 1.39)	0.97
75–79	43.8%	27.2%	2.09	(1.56, 2.81)	< 0.001
**Gender**					
Female	1.0%	0.5%	1.98	(0.42, 6.78)	0.32
Male	99.0%	99.5%	REF		
**Race**					
White	78.6%	78.9%	REF		
Black	14.7%	15.3%	0.92	(0.64, 1.30)	0.64
Other	6.7%	5.8%	1.11	(0.65, 1.81)	0.68
**Geographic Region**					
Northeast	10.9%	11.4%	REF		
Midwest	17.3%	24.9%	0.69	(0.44, 1.11)	0.12
West	18.8%	19.9%	0.83	(0.52, 1.33)	0.43
South	53.0%	43.9%	1.15	(0.78, 1.75)	0.50
**Weight, kg**					
61–69	13.1%	3.8%	3.76	(2.45, 5.70)	< 0.001
70–79	12.1%	10.7%	1.18	(0.79, 1.72)	0.41
80+	74.8%	85.4%	REF		
**Key Comorbid Conditions**^b^					
Heart Failure	34.8%	31.1%	1.12	(0.84, 1.47)	0.44
Hypertension	86.3%	84.8%	1.13	(0.74, 1.75)	0.59
Vascular Disease	31.0%	28.2%	1.16	(0.87, 1.53)	0.31
Diabetes	70.9%	70.1%	0.96	(0.70, 1.32)	0.80
Prior Bleeding	6.1%	4.7%	1.20	(0.69, 1.99)	0.50
Stroke	11.2%	9.0%	1.09	(0.72, 1.62)	0.66
**Creatinine Categories (%), in mg/dL**					
1.50–1.59	13.7%	23.2%	REF		
1.60–1.79	23.6%	31.0%	1.33	(0.88, 2.02)	0.18
≥1.80	62.6%	45.8%	2.51	(1.76, 3.69)	< 0.001
**Elixhauser Comorbidities**					
0–2	13.1%	12.3%	REF		
3–4	27.2%	32.8%	0.70	(0.44, 1.13)	0.14
≥ 5	55.0%	50.4%	0.82	(0.50, 1.38)	0.46

Model c statistic: 0.69

^a^Adjusted for all the other variables in the table

^b^For each condition, the reference category is patients without the condition

### Examining patients excluded from the Main analysis

A considerable proportion of patients who received a DOAC from the VA during the study period (33%) were excluded from this study, mainly because they received more than one DOAC or because they received more than one dose of a DOAC. We wished to explore this group further, to better understand the characteristics of such patients and the potential rates of guideline-discordant therapy among them, as opposed to the main study population. We will focus on patients who received rivaroxaban, since relatively few patients received dabigatran and the rules around apixaban dosing are extremely complicated.

Of the 25,656 patients who received any rivaroxaban prescription, 19,177 (75%) received only rivaroxaban, and only at one dose, and were thus included in our main analysis. Of the 6479 patients excluded from the study, 4027 (62%) received rivaroxaban and another DOAC, 1264 (20%) received both high and low doses of rivaroxaban, and 1188 (18%) received both doses of rivaroxaban and another DOAC. We will analyze the first two groups below; the third group is too complicated to analyze in detail.

Of the 4027 patients who received both rivaroxaban and another DOAC, 3423 (85%) received rivaroxaban and dabigatran, 98 (2%) received rivaroxaban and apixaban, and 506 (13%) received all three DOACs. A small number of patients (343, or 9%) did not have information on eGFR. Among the patients with eGFR information, 251 received a low dose of rivaroxaban; 42 (17%) of those inappropriately so and 209 of those (83%) appropriately so. Meanwhile, 3433 patients received a full dose of rivaroxaban, 130 of those (4%) inappropriately so and 3303 of those (96%) appropriately so. The rates of inappropriate dosing are similar in this population of excluded patients to our main analysis: in the main analysis, 23% of low doses were inappropriate, compared to 17% here. In the main analysis, 6% of full doses were inappropriate, compared to 4% here.

Of the 1264 patients who received both doses of rivaroxaban, 899 (71%) received the full dose first and 365 (29%) received the low dose first. Some of these patients (93 patients, or 7%), were missing information on eGFR. Of the patients who received the full dose first, 529 (59%) received this dose appropriately and 307 (41%) received it inappropriately. Of the patients who received the low dose first, 60 (16%) received this dose appropriately and 275 (84%) received it inappropriately.

## Discussion

In this study, we found that guideline-discordant dosing of DOACs is a fairly common issue in the VA healthcare system. During 2010–2016, 23% of patients who met criteria to receive low-dose therapy actually received full-dose therapy, and 6% of patients who met criteria to receive full-dose therapy actually received low-dose therapy. These phenomena varied widely by site of care, with some sites having almost no guideline-discordant dosing and others much higher rates than these average values. The proportion of guideline-discordant dosing improved somewhat over time, but only decreased by about 20% in a relative sense. Our data suggest that the proportion of guideline-discordant dosing for each medication was highest in the first year, and decreased thereafter. However, the absolute number of patients receiving guideline-discordant doses actually increased over time, as more and more patients received DOAC prescriptions each year. Our analysis of patients excluded from the study suggests that a large percentage of patients who received both full- and low-dose therapy initially received the guideline-discordant dose, which was then noticed and corrected. This implies that an even larger number of patients are exposed to a guideline-discordant dose than our main results would indicate, if only for a short time.

We also examined patient-level predictors of receiving guideline-discordant low-dose therapy. These predictors included some that may make sense on some level, such as older age, higher creatinine, or lower body mass. Still, it must be noted that every patient in our analyses was recommended for full-dose therapy based on FDA and VA guidelines. The rate of guideline-discordant dosing was highest with apixaban, which may in part reflect the complexity of dosing recommendations for this medication. Perhaps some providers found it mentally taxing to figure out which patients should receive which dose, or misinterpreted the guidelines to think that patients with only one out of three criteria should also receive a low dose.

Other findings are harder to explain. For example, we saw regional variations in terms of the use of guideline-discordant low-dose dabigatran, suggesting that a particular style of practice was in vogue in certain parts of the country. Still other findings seem counter-productive, such as providing guideline-discordant low-dose therapy to patients with higher BMI. Patients with higher BMI could be at even higher risk of thrombosis than normal-BMI patients when receiving a DOAC dose that is too low. Therefore, this practice seems particularly likely to harm patients.

At least two previous studies have examined the phenomenon of guideline-discordant DOAC dosing in NVAF. One study, which examined a prospective cohort of older patients from Massachusetts and Georgia, found that 15% of patients received an inappropriately high dose of DOACs and 5% an inappropriately low dose [6]. These numbers correspond fairly closely to our figures of 23 and 6%. Another study from Michigan found that many DOAC recipients do not undergo sufficient monitoring of renal function [5]. Our study has several important advantages over the earlier ones. We used data from a large, integrated healthcare system that spans all 50 states. Despite being an automated dataset which reflects a real-world patient population, our data included many details, such as lab values and prescribing data, that are not usually available in such a large dataset. The size of the dataset enabled us to profile 130 medical centers on their patterns of practice, and to examine changes in prescribing patterns by year. We also conducted separate analyses for each DOAC. Therefore, our report adds much new information.

We found that guideline-discordant dosing of DOACs was fairly common. However, several features of the VA system in fact may tend to minimize this sort of guideline discordant dosing. The VA has a large number of clinical pharmacists, many of whom are directly involved in managing anticoagulation therapy [12]. Many VA prescriptions for DOAC therapy may in fact be directly initiated or managed by clinical pharmacists, who may manage therapy more strictly according to VA and FDA guidelines than physicians would. The VA also has clear internal guidance about the use of many medications, including DOACs [7], and strong pharmacy structures at the national, regional, and medical center level that oversee and improve prescribing [13]. In fact, our analysis of patients excluded from the main analysis implies that many guideline-discordant doses were “caught” and changed to the guideline-concordant dose. However, we see from our study that even within such a system, a subset of prescriptions was not in line with VA and FDA recommendations. Outside of the context of a strong health system such as VA, the guideline-discordant prescribing that we observed here could be even more widespread, especially if the systems in place to correct guideline-discordant doses are weaker than those in the VA.

A question that arises from this study is what impact guideline-discordant dosing of DOACs has on patient outcomes. It seems likely that a too-low dose would lead to excess risk of thrombosis, while a too-high dose would lead to excess risk of bleeding. We plan to examine this issue in a future analysis.

This study benefited from a highly detailed and very large dataset, as well as the ability to compare 130 medical centers across the VA system. However, this study also has limitations. One of the most important is that we classified patients based on their most recent creatinine prior to their first DOAC prescription. It is possible that their creatinine was different on other occasions, and that this may have influenced the dose that would be prescribed to them. However, we believe it was reasonable to base our study on the most recent creatinine value, since clinicians should have looked at that value to inform their dosing decisions. Also, some concomitant medications, such as dronedarone, systemic ketoconazole, or Pgp-CYP3A4 inhibitors, require a DOAC dose adjustment [2–4]. We did not examine this issue. While the number of patients receiving such medications may be small, this could explain at least some of the patients who were apparently receiving a guideline-discordant low dose. In addition, although the guidelines do not mention it as an issue, other clinicians may have offered reduced doses because of concomitant anti-platelet medications. We also did not examine that, in part because aspirin is often purchased over the counter and therefore does not appear in our database.

Another limitation is that the VA population is mostly male and has a high burden of illness, possibly limiting its representativeness relative to the US population. Additionally, our study data only go through 2016; future studies should examine how prescribing has changed since the study period ended. In particular, we only report the first three years of data on apixaban prescribing after its approval. Finally, the VA healthcare system itself may not be typical of most US healthcare – which implies, as we said above, that guideline-discordant DOAC dosing may in fact be even more prevalent outside the VA system.

## Conclusions

In summary, a meaningful proportion of DOAC prescriptions within the VA system are inconsistent with the dosing recommendations set forth by the FDA and the VA’s own national pharmacy service. Most immediately, this represents an opportunity for the VA system to apply its considerable strengths toward ensuring that all prescriptions comply with clinical recommendations. However, we also plan to look into the impact that this guideline-discordant dosing has on patients’ outcomes. If this problem is even more prevalent outside VA, as we suspect it might be, a considerable number of patients could be harmed by inappropriate dosing of DOACs.

## Supplementary Information

**Additional file 1.** International Classification of Diseases (ICD) codes to define valvular heart disease, comorbid conditions, and stroke risk factors.

**Additional file 2.** Predictors of receiving full-dose DOAC therapy among patients recommended for low-dose therapy.

## Data Availability

The data that support the findings of this study are available from the Veterans Health Administration, but restrictions apply to the availability of these data, which were used under license for the current study, and so are not publicly available. Data are however available from the authors upon reasonable request and with permission of the Veterans Health Administration. Our statistical code is available upon request. Requests or inquiries should be directed to Dr. Rose at adamrose@bu.edu .

## Abbreviations

AOR: Adjusted Odds Ratio
BMI: Body Mass Index
DOAC: Direct-Acting Oral Anticoagulant
eGFR: Estimated Glomerular Filtration Rate
FDA: United States Food and Drug Administration
ICD: International Classification of Diseases (Diagnosis Codes)
NVAF: Non-Valvular Atrial Fibrillation
VA: Veterans Health Administration
VAMC: VA Medical Center
