# Supplementary material for: Guideline-discordant dosing of direct-acting oral anticoagulants in the veterans health administration
Source: BMC Health Serv Res. 2021 Dec 18;21:1351. doi: 10.1186/s12913-021-07397-x (PMC8684634; doi:10.1186/s12913-021-07397-x)
Supplement: Supplementary file 1 — Additional file 1. International Classification of Diseases (ICD) codes to define valvular heart disease, comorbid conditions, and stroke risk factors. [file 12913_2021_7397_MOESM1_ESM.docx]

Online Appendix A: International Classification of Diseases (ICD) codes to define valvular heart disease, comorbid conditions, and stroke risk factors.

|  | ICD-9-CM | ICD-10-CM |
| --- | --- | --- |
| Valvular Heart Disease – Diagnosis Codes | 394.0 Mitral stenosis  394.2 Mitral stenosis with insufficiency  396.1 Mitral stenosis with aortic insufficiency  396.8 Mitral and aortic multiple valvular disease  396.9 Mitral and aortic valve disease NOS  746.5 Congenital mitral stenosis  V43.3 Heart valve replacement NEC | I05.0 Rheumatic mitral stenosis  I05.2 Rheumatic mitral stenosis with insufficiency  I08.0 Rheumatic disorders of both mitral and aortic valves  I08.1 Rheumatic disorders of both mitral and tricuspid valves  I08.3 Combined rheumatic disorders of mitral, aortic, and tricuspid valves  I08.8 Other rheumatic multiple valve diseases  I08.9 Rheumatic multiple valve disease, unspecified  I09.81 Rheumatic heart failure  I34.2 Nonrheumatic mitral valve stenosis  Q23.2 Congenital mitral stenosis  Q23.8 Other congenital malformations of aortic and mitral valves  Q23.9 Congenital malformations of aortic and mitral valves, unspecified  Z95.2 Presence of prosthetic heart valve |
| Valvular Heart Disease – Procedure Codes | 35.02 Closed mitral valvotomy  35.12 Open mitral valvuloplasty  35.20 Replace heart valve NOS  35.22 Replace aortic valve NEC  35.24 Replace mitral valve NEC  35.26 Replace pulmonary valve NEC  35.28 Replace tricuspid valve NEC | 02QG Repair of mitral valve  02RF Replacement of aortic valve  02RG Replacement of mitral valve  02RH Replacement of pulmonary valve  02RJ Replacement of tricuspid valve |
| Heart Failure | 398.91, 402.x, 404.01, 404.11, 404.03, 428.x | I42.9, I50.x |
| Hypertension | 401.x, 402.x, 403.x, 404.x, 405.x, 437.2 | I50.30, I50.40, I50.9, N03.9, N18.1, N18.2, N18.3, N18.4, N18.5, N18.6, N18.9, N19, Z99.2, I10, I11, I11.0, I11.9, I12, I12.0, I12.9, I13, I13.0, I13.1, I13.10, I13.11, I13.2, I15, I15.0, I15.1, I15.2, I15.8, I15.9, I16, I16.0, I16.1, I16.9 |
| Vascular Disease | 410.x, 411.x, 412, 440.x, 441.x, 443.1, 443.89 | H91.90, G40.909, E11.9, N28.9, I21.x-I24.x, I70.x |
| Diabetes | 249.x, 250.x, 357.2, 362, 366.41 | B35.1, E03.9, E23.2, E27.49, E66.9, E78.1, E78.6, G56.00, H21.1X9, H33.40, H34.9, H35.049, H40.9, H42, H43.10, H47.099, H47.20, H54.0, H54.10, H54.7, H91.90, I10, I12.0, I12.9, I70.209, K31.84, L03.039, L03.119, L89.509, L89.609, L97.209, L97.309, L97.409, L97.509, L97.519, L97.529, L97.909, L97.919, L97.929, L98.499, M54.14, M54.16, M86.9, N18.1, N18.2, N18.3, N18.4, N18.5, N18.6, N18.9, N52.1, R19.7, R80.9, Z79.4, Z99.2, E08-E13 |
| Prior Bleeding | 423.0, 430, 431, 432.x, 455.2, 455.5, 455.8, 456.0, 456.2, 459.0, 530.7, 530.82, 531.01, 531.41, 531.61, 532.01, 532.21, 532.41, 532.61, 533.21, 533.4, 534.41, 535.01, 535.11, 535.31, 535.41, 535.51, 535.61, 537.83, 537.84, 562.02, 562.03, 562.12, 562.13, 568.81, 569.3, 569.85, 578.x, 596.7, 599.7, 719.1x, 782.7, 784.7, 784.8, 786.3 | I312, I609, I619, I621, I6200, I629, K648, K644, K648, I8501, I8511, R58, K226, K228, K250, K254, K256, K260, K262, K264, K266, K272, K274, K284, K2901, K2941, K2951, K2941, K2951, K2961, K2971, K2991, K2981, K31811, K3182, K5711, K5713, K5731, K5733, K661, K625, K5521, K920, K921, K922, N3289, R319, R310, R312, R311, M2500, M25019, M25029, M25039, M25049, M25059, M25069, M25073, M25076, M2508, M2500, R233, R040, R041, R042, R049 |
| Prior Stroke | 433.01, 433.1, 433.11, 433.21, 433.31, 433.81, 433.91, 434.01, 434.11, 434.91, 435.x, 436 | G93.49, I67.89, G45.x, I63.x, I74.x |

ICD-9 CM: International Classification of Diseases, Clinical Modification – 9^th^ Edition

ICD-10 CM: International Classification of Diseases, Clinical Modification – 10^th^ Edition
