# Supplementary material for: Guideline-discordant dosing of direct-acting oral anticoagulants in the veterans health administration
Source: BMC Health Serv Res. 2021 Dec 18;21:1351. doi: 10.1186/s12913-021-07397-x (PMC8684634; doi:10.1186/s12913-021-07397-x)
Supplement: Supplementary file 2 — Additional file 2. Predictors of receiving full-dose DOAC therapy among patients recommended for low-dose therapy. [file 12913_2021_7397_MOESM2_ESM.docx]

Online Appendix B: Predictors of receiving full-dose DOAC therapy among patients recommended for low-dose therapy.

Table B1: Patient-level factors associated with receiving full-dose dabigatran from the Veterans Health Administration, as compared to receiving low-dose, among those who met clinical criteria to receive low-dose dabigatran for non-valvular atrial fibrillation (n = 247).

| Characteristic | Full-Dose  (n = 173) | Low-Dose  (n = 74) | Adjusted Odds Ratio* to Receive Full-dose DOAC | 95% Confidence Interval | p-value |
| --- | --- | --- | --- | --- | --- |
| **Age (yrs)** |  |  |  |  |  |
| ≤ 69 | 23.1% | 10.8% | REF |  |  |
| 70-74 | 15.6% | 2.7% | 2.69 | (0.56, 19.89) | 0.26 |
| 75-79 | 16.2% | 10.8% | 0.72 | (0.21, 2.50) | 0.60 |
| ≥ 80 | 45.1% | 75.7% | 0.18 | (0.09, 0.75) | 0.02 |
| **Gender** |  |  |  |  |  |
| Female | 2.3% | 6.8% | 0.33 | (0.06, 1.52) | 0.16 |
| Male | 97.7% | 93.2% | REF |  |  |
| **Race** |  |  |  |  |  |
| White | 80.3% | 78.4% | REF |  |  |
| Black | 13.9% | 14.9% | 0.51 | (0.20, 1.35) | 0.17 |
| Other | 5.8% | 6.8% | 0.53 | (0.13, 2.37) | 0.38 |
| **Geographic Region** |  |  |  |  |  |
| Northeast | 13.9% | 14.9% | REF |  |  |
| Midwest | 24.3% | 23.0% | 0.91 | (0.31, 2.65) | 0.86 |
| West | 24.9% | 21.6% | 0.79 | (0.26, 2.31) | 0.66 |
| South | 37.0% | 40.5% | 0.66 | (0.24, 1.74) | 0.41 |
| **BMI, kg/m2** |  |  |  |  |  |
| < 25 | 51.4% | 54.1% | REF |  |  |
| 25-29.9 | 25.4% | 32.4% | 0.83 | (0.40, 1.72) | 0.61 |
| 30-34.9 | 11.6% | 8.1% | 1.58 | (0.48, 6.24) | 0.48 |
| ≥ 35 | 9.8% | 2.7% | 1.90 | (0.40, 14.26) | 0.46 |
| **Key Comorbid Conditions†** |  |  |  |  |  |
| Heart Failure | 29.5% | 27.0% | 0.78 | (0.36, 1.68) | 0.52 |
| Hypertension | 71.7% | 78.4% | 0.44 | (0.17, 1.08) | 0.08 |
| Vascular Disease | 14.4% | 20.3% | 0.60 | (0.24, 1.58) | 0.29 |
| Diabetes | 50.9% | 44.6% | 0.70 | (0.34, 1.44) | 0.34 |
| Prior Bleeding | 1.7% | 5.4% | 0.23 | (0.03, 1.59) | 0.14 |
| Stroke | 12.7% | 10.8% | 1.54 | (0.55, 4.67) | 0.42 |
| **eGFR Categories (%), in units of mL/min/1.73 m^2^** |  |  |  |  |  |
| < 20 | 23.1% | 21.6% | REF |  |  |
| 20-29 | 76.9% | 78.4% | 1.17 | (0.53, 2.54) | 0.70 |
| **Elixhauser Comorbidities** |  |  |  |  |  |
| 0-2 | 20.2% | 20.3% | REF |  |  |
| 3-4 | 31.2% | 35.1% | 1.44 | (0.52, 3.98) | 0.48 |
| ≥ 5 | 43.9% | 35.1% | 2.12 | (0.69, 6.63) | 0.19 |

Model c statistic: 0.74

*Adjusted for all the other variables in the table.

†For each condition, the reference category is patients without the condition.

Table B2: Patient-level factors associated with receiving full-dose rivaroxaban from the Veterans Health Administration, as compared to receiving low-dose, among those who met clinical criteria to receive low-dose rivaroxaban for non-valvular atrial fibrillation (n = 2,346).

| Characteristic | Full-Dose  (n = 657) | Low-Dose  (n = 1,689) | Adjusted Odds Ratio* to Receive Full-dose DOAC | 95% Confidence Interval | p-value |
| --- | --- | --- | --- | --- | --- |
| **Age (yrs)** |  |  |  |  |  |
| ≤ 69 | 19.9% | 8.4% | REF |  |  |
| 70-74 | 14.6% | 8.1% | 0.68 | (0.44, 0.99) | 0.045 |
| 75-79 | 15.8% | 11.8% | 0.51 | (0.36, 0.74) | < 0.001 |
| ≥ 80 | 49.6% | 71.7% | 0.26 | (0.19, 0.35) | < 0.001 |
| **Gender** |  |  |  |  |  |
| Female | 2.7% | 1.8% | 1.27 | (0.63, 2.45) | 0.49 |
| Male | 97.3% | 98.2% | REF |  |  |
| **Race** |  |  |  |  |  |
| White | 82.6% | 86.9% | REF |  |  |
| Black | 11.6% | 7.1% | 1.23 | (0.86, 1.74) | 0.25 |
| Other | 5.8% | 6.0% | 0.97 | (0.63, 1.47) | 0.88 |
| **Geographic Region** |  |  |  |  |  |
| Northeast | 15.2% | 18.2% | REF |  |  |
| Midwest | 22.8% | 25.5% | 0.96 | (0.69, 1.34) | 0.82 |
| West | 18.9% | 17.0% | 1.22 | (0.87, 1.73) | 0.26 |
| South | 43.1% | 39.3% | 1.2 | (0.89, 1.63) | 0.23 |
| **BMI, kg/m2** |  |  |  |  |  |
| < 25 | 48.4% | 41.4% | REF |  |  |
| 25-29.9 | 35.0% | 40.7% | 0.66 | (0.53, 0.83) | < 0.001 |
| 30-34.9 | 9.9% | 12.0% | 0.58 | (0.41, 0.81) | 0.002 |
| ≥ 35 | 5.0% | 3.6% | 0.78 | (0.48, 1.26) | 0.32 |
| **Key Comorbid Conditions†** |  |  |  |  |  |
| Heart Failure | 20.8% | 18.2% | 1.16 | (0.88, 1.52) | 0.29 |
| Hypertension | 76.3% | 78.4% | 0.94 | (0.72, 1.24) | 0.65 |
| Vascular Disease | 20.4% | 15.8% | 1.33 | (1.02, 1.74) | 0.04 |
| Diabetes | 48.9% | 47.0% | 1.02 | (0.82, 1.27) | 0.86 |
| Prior Bleeding | 3.6% | 2.9% | 1.22 | (0.71, 2.08) | 0.46 |
| Stroke | 8.2% | 7.7% | 1.02 | (0.70, 1.47) | 0.92 |
| **eGFR Categories (%), in units of mL/min/1.73 m^2^** |  |  |  |  |  |
| < 30 | 9.1% | 13.6% | REF |  |  |
| 30-39 | 18.9% | 33.3% | 0.93 | (0.65, 1.35) | 0.69 |
| 40-49 | 72.0% | 53.2% | 2.26 | (1.63, 3.17) | < 0.001 |
| **Elixhauser Comorbidities** |  |  |  |  |  |
| 0-2 | 25.3% | 26.1% | REF |  |  |
| 3-4 | 38.8% | 37.5% | 0.97 | (0.74, 1.28) | 0.83 |
| ≥ 5 | 29.8% | 28.8% | 0.69 | (0.49, 0.98) | 0.04 |

Model c statistic: 0.69

*Adjusted for all the other variables in the table.

†For each condition, the reference category is patients without the condition.

Table B3: Patient-level factors associated with receiving full-dose apixaban from the Veterans Health Administration, as compared to receiving low-dose, among those who met clinical criteria to receive low-dose apixaban for non-valvular atrial fibrillation. This table includes patients with all three of the factors suggesting a need for low-dose therapy (i.e., age ≥ 80, serum creatinine ≥ 1.5, and body mass < 60 kg, n = 97). Because only two patients received full-dose therapy, it was not possible to perform statistical analyses; therefore, we tabulate the number of patients in each group.

| Characteristic | Full-Dose  (n = 2) | Low-Dose  (n = 95) |
| --- | --- | --- |
| **Age (yrs)** |  |  |
| 80-84 | 1 | 35 |
| 85-89 | 1 | 32 |
| 90+ | 0 | 28 |
| **Gender** |  |  |
| Female | 0 | 8 |
| Male | 2 | 87 |
| **Race** |  |  |
| White | 2 | 75 |
| Black | 0 | 7 |
| Other | 0 | 13 |
| **Geographic Region** |  |  |
| Northeast | 1 | 19 |
| Midwest | 0 | 16 |
| West | 0 | 19 |
| South | 1 | 41 |
| **Weight, kg** |  |  |
| <50 | 0 | 7 |
| 50-59 | 2 | 88 |
| **Key Comorbid Conditions** |  |  |
| Heart Failure | 0 | 20 |
| Hypertension | 1 | 75 |
| Vascular Disease | 0 | 13 |
| Diabetes | 0 | 39 |
| Prior Bleeding | 0 | 3 |
| Stroke | 0 | 16 |
| **Creatinine Categories (%), in mg/dL** |  |  |
| 1.50-1.59 | 0 | 25 |
| 1.60-1.79 | 1 | 32 |
| ≥1.80 | 1 | 38 |
| **Elixhauser Comorbidities** |  |  |
| 0-2 | 0 | 21 |
| 3-4 | 2 | 34 |
| ≥ 5 | 0 | 34 |

Table B4: Patient-level factors associated with receiving full-dose apixaban from the Veterans Health Administration, as compared to receiving low-dose, among those who met clinical criteria to receive low-dose apixaban for non-valvular atrial fibrillation. This table includes patients with two out of three of the factors suggesting a need for low-dose therapy (age ≥ 80, body mass < 60 kg, and serum creatinine < 1.5, n = 484).

| Characteristic | Full-Dose  (n = 85) | Low-Dose  (n = 399) | Adjusted Odds Ratio* to Receive Full-dose DOAC | 95% Confidence Interval | p-value |
| --- | --- | --- | --- | --- | --- |
| **Age (yrs)** |  |  |  |  |  |
| 80-84 | 47.1% | 32.1% | REF |  |  |
| 85-89 | 35.3% | 36.1% | 0.86 | (0.45, 1.64) | 0.64 |
| 90+ | 17.6% | 31.8% | 0.54 | (0.24, 1.18) | 0.13 |
| **Gender** |  |  |  |  |  |
| Female | 4.7% | 12.5% | 0.31 | (0.07, 1.00) | 0.08 |
| Male | 95.3% | 87.5% | REF |  |  |
| **Race** |  |  |  |  |  |
| White | 85.9% | 85.2% | REF |  |  |
| Black | 4.7% | 3.8% | 1.20 | (0.25, 4.41) | 0.80 |
| Other | 9.4% | 11.0% | 0.82 | (0.30, 2.02) | 0.68 |
| **Geographic Region** |  |  |  |  |  |
| Northeast | 10.6% | 16.0% | REF |  |  |
| Midwest | 18.8% | 19.0% | 2.05 | (0.74, 6.10) | 0.18 |
| West | 35.3% | 25.6% | 2.68 | (1.05, 7.59) | 0.048 |
| South | 35.3% | 39.3% | 1.45 | (0.57, 4.04) | 0.45 |
| **Weight, kg** |  |  |  |  |  |
| <50 | 8.2% | 13.0% | 0.88 | (0.31, 2.20) | 0.79 |
| 50-59 | 91.8% | 87.0% | REF |  |  |
| **Key Comorbid Conditions†** |  |  |  |  |  |
| Heart Failure | 20.0% | 15.3% | 1.41 | (0.64, 2.98) | 0.38 |
| Hypertension | 71.8% | 66.9% | 1.17 | (0.58, 2.39) | 0.67 |
| Vascular Disease | 15.3% | 14.0% | 0.88 | (0.37, 2.01) | 0.76 |
| Diabetes | 35.3% | 35.1% | 0.81 | (0.42, 1.53) | 0.51 |
| Prior Bleeding | 1.2% | 5.3% | 0.27 | (0.02, 1.46) | 0.22 |
| Stroke | 14.1% | 12.3% | 0.70 | (0.26, 1.66) | 0.44 |
| **Creatinine Categories (%), in mg/dL** |  |  |  |  |  |
| < 1 | 56.5% | 43.4% | REF |  |  |
| 1.0-1.29 | 24.7% | 25.1% | 0.82 | (0.43, 1.53) | 0.54 |
| 1.3-1.49 | 4.7% | 16.8% | 0.16 | (0.04, 0.49) | 0.004 |
| **Elixhauser Comorbidities** |  |  |  |  |  |
| 0-2 | 24.7% | 30.6% | REF |  |  |
| 3-4 | 41.2% | 41.4% | 1.06 | (0.50, 2.32) | 0.87 |
| ≥ 5 | 22.4% | 19.8% | 1.07 | (0.37, 3.04) | 0.90 |

Model c statistic: 0.70

*Adjusted for all the other variables in the table.

†For each condition, the reference category is patients without the condition.

Table B5: Patient-level factors associated with receiving full-dose apixaban from the Veterans Health Administration, as compared to receiving low-dose, among those who met clinical criteria to receive low-dose apixaban for non-valvular atrial fibrillation. This table includes patients with two out of three of the factors suggesting a need for low-dose therapy (serum creatinine ≥ 1.5, body mass < 60 kg, and age < 80, n = 30). Because only thirty patients were in the entire analysis, it was not possible to perform statistical analyses; therefore, we tabulate the number of patients in each group.

| Characteristic | Full-Dose  (n = 12) | Low-Dose  (n = 18) |
| --- | --- | --- |
| **Age (yrs)** |  |  |
| ≤ 69 | 5 | 6 |
| 70-74 | 2 | 3 |
| 75-79 | 5 | 9 |
| **Gender** |  |  |
| Female | 0 | 0 |
| Male | 12 | 18 |
| **Race** |  |  |
| White | 9 | 12 |
| Black | 2 | 4 |
| Other | 1 | 2 |
| **Geographic Region** |  |  |
| Northeast | 0 | 2 |
| Midwest | 4 | 3 |
| West | 2 | 4 |
| South | 6 | 9 |
| **Weight, kg** |  |  |
| <50 | 1 | 3 |
| 50-59 | 11 | 15 |
| **Key Comorbid Conditions†** |  |  |
| Heart Failure | 4 | 6 |
| Hypertension | 10 | 16 |
| Vascular Disease | 6 | 5 |
| Diabetes | 6 | 7 |
| Prior Bleeding | 2 | 1 |
| Stroke | 0 | 5 |
| **Creatinine Categories (%), in mg/dL** |  |  |
| 1.50-1.59 | 4 | 6 |
| 1.60-1.79 | 4 | 4 |
| ≥1.80 | 4 | 8 |
| **Elixhauser Comorbidities** |  |  |
| 0-2 | 1 | 0 |
| 3-4 | 4 | 4 |
| ≥ 5 | 7 | 13 |

Table B6: Patient-level factors associated with receiving full-dose apixaban from the Veterans Health Administration, as compared to receiving low-dose, among those who met clinical criteria to receive low-dose apixaban for non-valvular atrial fibrillation. This table includes patients with two out of three of the factors suggesting a need for low-dose therapy (i.e., age ≥ 80, serum creatinine ≥ 1.5, and body mass ≥ 60 kg, n = 2,639).

| Characteristic | Full-Dose  (n = 398) | Low-Dose  (n = 2,231) | Adjusted Odds Ratio* to Receive Full-dose DOAC | 95% Confidence Interval | p-value |
| --- | --- | --- | --- | --- | --- |
| **Age (yrs)** |  |  |  |  |  |
| 80-84 | 52.0% | 44.4% | REF |  |  |
| 85-89 | 32.4% | 35.6% | 0.72 | (0.56, 0.94) | 0.02 |
| 90+ | 15.6% | 19.9% | 0.66 | (0.47, 0.92) | 0.02 |
| **Gender** |  |  |  |  |  |
| Female | 0.5% | 0.5% | 1.51 | (0.22, 6.49) | 0.62 |
| Male | 99.5% | 99.5% | REF |  |  |
| **Race** |  |  |  |  |  |
| White | 90.7% | 87.5% | REF |  |  |
| Black | 5.0% | 7.2% | 0.71 | (0.41, 1.16) | 0.19 |
| Other | 4.3% | 5.3% | 0.82 | (0.45, 1.40) | 0.48 |
| **Geographic Region** |  |  |  |  |  |
| Northeast | 15.3% | 17.3% | REF |  |  |
| Midwest | 26.6% | 24.4% | 1.21 | (0.84, 1.75) | 0.32 |
| West | 23.1% | 18.9% | 1.36 | (0.93, 2.00) | 0.12 |
| South | 34.9% | 39.4% | 0.95 | (0.67, 1.36) | 0.77 |
| **Weight, kg** |  |  |  |  |  |
| 61-69 | 12.3% | 14.5% | 0.71 | (0.48, 1.03) | 0.08 |
| 70-79 | 25.9% | 27.0% | 0.97 | (0.73, 1.27) | 0.80 |
| 80+ | 61.8% | 58.5% | REF |  |  |
| **Key Comorbid Conditions†** |  |  |  |  |  |
| Heart Failure | 20.4% | 21.1% | 1.17 | (0.86, 1.59) | 0.31 |
| Hypertension | 79.4% | 83.5% | 1.00 | (0.71, 1.41) | 0.98 |
| Vascular Disease | 22.6% | 20.3% | 1.30 | (0.96, 1.74) | 0.09 |
| Diabetes | 52.5% | 57.5% | 0.82 | (0.63, 1.07) | 0.14 |
| Prior Bleeding | 6.5% | 3.5% | 1.90 | (1.14, 3.08) | 0.01 |
| Stroke | 9.5% | 7.5% | 1.58 | (1.05, 2.33) | 0.02 |
| **Creatinine Categories (%), in mg/dL** |  |  |  |  |  |
| 1.50-1.59 | 42.5% | 18.6% | REF |  |  |
| 1.60-1.79 | 31.9% | 32.7% | 0.42 | (0.32, 0.55) | < 0.001 |
| ≥1.80 | 25.6% | 48.7% | 0.22 | (0.17, 0.30) | < 0.001 |
| **Elixhauser Comorbidities** |  |  |  |  |  |
| 0-2 | 24.1% | 19.6% | REF |  |  |
| 3-4 | 41.0% | 37.8% | 0.91 | (0.65, 1.27) | 0.58 |
| ≥ 5 | 26.9% | 34.8% | 0.67 | (0.45, 1.01) | 0.053 |

Model c statistic: 0.70

*Adjusted for all the other variables in the table.

†For each condition, the reference category is patients without the condition.
